# Design and modification of EGF4KDEL 7Mut, a novel bispecific ligand-directed toxin, with decreased immunogenicity and potent anti-mesothelioma activity

**DOI:** 10.1038/sj.bjc.6605297

**Published:** 2009-09-29

**Authors:** B J Stish, S Oh, H Chen, A Z Dudek, R A Kratzke, D A Vallera

**Affiliations:** 1 Department of Therapeutic Radiology-Radiation Oncology, Section on Molecular Cancer Therapeutics, Masonic Cancer Center, University of Minnesota, Minneapolis, MN, USA; 2 Department of Medicine, University of Minnesota, Minneapolis, MN, USA

**Keywords:** mesothelioma, EGF, IL-4, molecular therapeutics, nude mice, immunotoxin

## Abstract

**Background:**

Potency, immunogenicity, and toxicity are three problems that limit the use of targeted toxins in solid tumour therapy.

**Methods:**

To address potency, we used genetic engineering to develop a novel bispecific ligand-directed toxin (BLT) called EGF4KDEL, a novel recombinant anti-mesothelioma agent created by linking human epidermal growth factor (EGF) and interleukin-4 (IL-4) to truncated pseudomonas exotoxin (PE38) on the same single-chain molecule. Immunogenicity was reduced by mutating seven immunodominant B-cell epitopes on the PE38 molecule to create a new agent, EGF4KDEL 7Mut.

**Results:**

*In vitro*, bispecific EGF4KDEL showed superior anti-mesothelioma activity compared with its monospecific counterparts. Toxicity in mice was diminished by having both ligands on the same molecule, allowing administration of a 10-fold greater dose of BLT than a mixture of monomeric IL4KDEL and EGFKDEL. EGF4KDEL 7Mut, retained all of its functional activity and induced about 87% fewer anti-toxin antibodies than mice given the parental, non-mutated form. *In vivo*, intraperitoneal (IP) injection of the BLT showed significant (*P*<0.01) and impressive effects against two aggressive, malignant IP mesothelioma models when treatment was begun 14–16 days post tumour innoculation.

**Conclusion:**

These data show that EGF4KDEL 7Mut is a promising new anti-mesothelioma agent that was developed to specifically address the obstacles facing clinical utility of targeted toxins.

Malignant mesothelioma (MM) is a highly aggressive cancer of the serosal surfaces of the body with an invariably poor prognosis (Zucali and Giaccone, 2006). Owing to the inherent properties of MM, surgical resection or radiotherapeutic intervention are often not viable treatment options (Palumbo *et al*, 2008). Combination therapy with pemetrexed and cisplatin, the current standard of therapy for nonresectable MM, results in a median survival time of only 12.1 months and has many treatment-associated side effects (Vogelzang *et al*, 2003; Simon *et al*, 2008). With 15 000–20 000 deaths per year worldwide due to MM, new and improved therapies are desperately needed (Zervos *et al*, 2008).

Specifically targeting overexpressed cell surface receptors on tumour cells offers a promising alternative approach for MM therapy (Hassan *et al*, 2007). Ligand-directed cytotoxins containing a truncated pseudomonas exotoxin A (PE38) are a unique class of receptor-targeted biological drugs with extremely potent anti-cancer activity (Kreitman and Pastan, 1998). PE38 is used for cytotoxin construction because a single molecule delivered to the cytosol is sufficient to bring about cell killing by catalysing ADP ribosylation of elongation factor 2 (EF-2) leading to irreversible inhibition of protein synthesis and cell death (Pastan *et al*, 1992). Further studies have shown that modifying PE38 by adding a Lys-Asp-Glu-Leu (KDEL) C-terminal signal that prevents secretion of luminal endoplasmic reticulum (ER) proteins increased ER accumulation and enhanced potency of targeted toxins (Kreitman and Pastan, 1995). The advantage of ligand-directed toxins is that PE38KDEL-mediated cytotoxicity requires specific ligand-receptor binding and subsequent internalisation. This allows maximal anti-cancer activity while limiting collateral killing of normal cells.

Previous research has shown overexpression of epidermal growth factor receptor (EGFR) on most MM cell lines and clinical tumour samples (Cole *et al*, 2005; Govindan *et al*, 2005). Activation of EGFR promotes processes responsible for tumour growth and progression, including proliferation and maturation, angiogenesis, invasion, metastasis, and inhibition of apoptosis, making it a popular target for anti-cancer therapy (Yarden and Sliwkowski, 2001). Immunohistochemistry studies have also shown high expression levels of interleukin-4 receptor (IL-4R) on patient mesothelioma samples (Beseth *et al*, 2004). Interleukin-4 is an important cytokine in Th2 differentiation of T cells and IL-4R has proven to be a promising target for treatment of pancreatic cancer and glioblastoma (Rand *et al*, 2000; Shimamura *et al*, 2007). To enhance the activity of cytotoxins targeting IL-4R, a circularly permuted IL-4 cytotoxin has been described that improved binding site exposure without altering ligand-receptor specificity (Kreitman *et al*, 1994) and these alterations were incorportated into EGF4KDEL. Both EGFR and IL-4R are ideal targets for ligand-directed toxin therapies as both are cell surface molecules that internalise rapidly upon ligand binding (Haigler *et al*, 1978; Kawakami *et al*, 2002a).

Targeting solid tumours with ligand-directed cytotoxins has been hampered by problems with immunogenicity as effective therapy often requires multiple treatments resulting in the generation of neutralising antibodies, mostly against the PE38 portion of the molecule (Hassan *et al*, 2004). A potential solution to this problem was recently published by Onda and Pastan who showed that there are only seven major B-cell epitopes on the PE38 molecule (Onda *et al*, 2006). Subsequently, they described a series of mutations that did not alter the catalytic activity of PE38 yet nearly eliminated the immune response of mice (Onda *et al*, 2008). In these studies, they show that the mouse is a valid model of anti-toxin immunogenicity.

We have synthesised a unique class of single-chain biological agents called bispecific ligand-directed toxin (BLT) with significant advantages over traditional monospecific ligand-directed toxins including increased potency, broader tumour targeting, and decreased toxicity (Vallera *et al*, 2005b, 2008, 2009; Stish *et al*, 2007a, 2007b, 2008). In this study, we reveal for the first time that EGF4KDEL, a BLT targeting both EGFR and IL-4R, shows potent activity against MM both *in vitro* and *in vivo* and has significantly reduced toxicity when given therapeutically. Furthermore, we show the benefit of readily modifiable biological agents by synthesising a genetically mutated form of EGF4KDEL with decreased immunogenicity. This new molecule, EGF4KDEL 7Mut, retained the ability to selectively kill MM tumours while producing a significantly decreased neutralising antibody response when injected into Balb/c mice. Our research shows significant anti-cancer properties of EGF4KDEL 7Mut and its potential for clinical treatment of mesothelioma.

## Materials and methods

### Construction of EGF4KDEL and EGF4KDEL 7Mut

**Figure 1:**
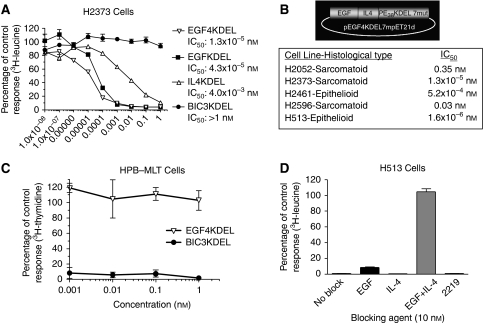
*In vitro* cytotoxicity and specificity of EGF4KDEL. (**A**) The activity of EGF4KDEL was tested against the human MM cell line H2373 in a protein synthesis inhibition assay. The effects of EGF4KDEL, EGFKDEL, IL4KDEL, and the T-cell targeting BIC3KDEL were determined by analysing ^3^H-leucine uptake by H2373 cells following a 72-h incubation with each agent. Data are reported as percent control response as determined by (^3^H-leucine activity of treated cells/^3^H-leucine activity of H2373 cells in media alone) × 100. Each data point represents an average of triplicate measures±s.d. (**B**) Effect of EGF4KDEL on a panel of different human MM cell lines as measured by protein synthesis inhibition assay. IC_50_ values represent the concentration required to inhibit 50% of protein synthesis compared with untreated controls. A cartoon showing the construction of the EGF4KDEL 7mut gene is also shown. (**C**) *In vitro* specificity of EGF4KDEL was shown by testing its activity towards EGFR^−^/IL-4R^−^ HPB-MLT leukaemic T cells in a ^3^H-thymidine incorporation assay. BIC3KDEL was included as a positive control. (**D**) A blocking assay was performed to prove the functionality of each fragment of the EGF4KDEL molecule. H513 cells were incubated with 0.01 nM EGF4KDEL and the effect of blocking with a 10 nM concentration of recombinant EGF and IL-4, both separately and in combination was determined by measuring protein synthesis. The non-binding bispecific recombinant 2219 scFv molecule was included as a specificity control. Data is presented as a mean of triplicate measures±s.d. and was calculated as percentage of protein synthesis relative to cells incubated with antibodies alone.

Synthesis and assembly of hybrid genes encoding the single-chain EGF4KDEL was accomplished using DNA-shuffling and DNA-cloning techniques. The fully assembled fusion gene (from 5′ end to 3′ end) consisted of an *Nco*1 restriction site, an ATG initiation codon, the genes for human EGF and circularly permutated human IL-4 linked by a 20 amino-acid segment of human muscle aldolase (HMA), the 7 amino-acid EASGGPE linker and the first 362 amino acids of the PE molecule with KDEL replacing the REDLK at the C terminus, a *Not*I restriction site at the 3′ end of the construct. The HMA segment was incorporated into the molecule as a flexible, non-immunogenic linker (Vallera *et al*, 2005b). The gene-encoding circularly permuted IL-4 was generously provided by Drs RJ Kreitman and I Pastan (NIH, Bethesda MD, USA). The resultant 1752 bp *Nco*I/*Not*II fragment gene was spliced into the pET21d bacteria expression vector under control of an isopropyl-b-D-thiogalactopyranoside inducible T7 promoter (Figure 1). DNA sequencing analysis (Biomedical Genomics Center, University of Minnesota) was used to verify that the gene was correct in sequence and had been cloned in frame. To create an EGF4KDEL molecule with decreased immunogenicity, eight amino acids representing the seven major epitopes on PE38 (Onda *et al*, 2008) were mutated using the QuickChange Site-Directed Mutagenesis Kit (Stratagene. La Jolla CA, USA). The following amino acids were altered: R490A, R513A, R467A, E548S, K590S, R432G, Q332S, R313A and confirmed with DNA sequencing. Genes for monospecific cytotoxins splicing mutated PE38(KDEL) to human EGF (EGFKDEL) and mutated human IL-4 (cpIL4KDEL) were created using the same techniques. BIC3KDEL, a bispecific immunotoxin-targeting T-cell surface marker CD3, was made by replacing the DT_390_ portion of the BIC3 molecule described previously with PE38(KDEL) (Vallera *et al*, 2005a).

### Isolation of inclusion bodies, refolding and purification

Proteins were produced as described previously (Vallera *et al*, 2008) with some minor modifications to improve yield and purity. Then, 10 mg ml^−1^ of dithierythritol was included in refolding buffer to decrease protein aggregation. In addition, refolded protein was directly diluted instead of being dialysed before loading onto an ion exchange column. Finally, the purity of protein isolated from the ion exchange column was further enhanced using an FPLC and Supradex 200 size exclusion column (Sigma, Ronconcoma, NY, USA). This modified protocol resulted in a yield of 5–10 mg of protein per litre of culture and a final product with >95% purity.

### Cell culture

The human malignant mesothelioma cell lines H2373, H513, and H2461, as well as the SV40-transformed normal mesothelial cell line MeT-5A were obtained from American Type Culture Collection (ATCC, Rockville, MD, USA) and have been described previously (Pass *et al*, 1995). MM cell lines were grown as monolayers in RPMI-1640 media (Cambrex, East Rutherford, NJ, USA) supplemented with 10% fetal bovine serum, 2 mmol l^−1^ L-glutamine, 100 units ml^−1^ penicillin, and 100 *μ*g ml^−1^ streptomycin. MeT-5A cells were cultured in Medium 199 supplemented with 10% FBS, 2 mmol l^−1^ L-glutamine, 3.3 nM EGF, 400 nM hydrocortisone, and 870 nM insulin. LP-9, a normal non-immortalized human mesothelial cell line, was obtained from the NIA Aging Cell Culture Repository at the Coriell Institute (Camden, NJ, USA) and was maintained in a Ham's F12/Medium 199 mixture supplemented with 10% fetal bovine serum, 2 mmol l^−1^ L-glutamine, 0.4 *μ*g ml^−1^ hydrocortisone, and 10 ng ml^−1^ epidermal growth factor. HPB–MLT, a human T-cell leukaemia cell line, was used as a negative control and has been described previously (Vallera *et al*, 2005a).

For *in vivo* experiments, H2373 and H513 cells were stably transfected with dual vectors containing both the firefly luciferase (Luc) and green fluorescent protein (GFP) genes, as well as a blastocidin resistance gene (Clontech Laboratories, Mountain View, CA, USA). Transfection was performed with Lipofectamine reagent (Invitrogen, Carlsbad, CA, USA) and stable clones were established using a FACSDiva flow cytometer (University of Minnesota Flow Cytometry Core Facility of the Masonic Cancer Center) to seed individual GFP-positive cells into a 96-well plate. Each clone used in this study, H2373/Luc/GFP and H513/Luc/GFP, retained identical morphological and biological properties to the specific parental cell line.

### Bioassays to measure *in vitro* protein synthesis inhibition

To determine the effect of EGF4KDEL on malignant mesothelioma cells, assays measuring protein synthesis via ^3^H-leucine incorporation were conducted. Cells (10^4^ per well) were added to a 96-well flat-bottomed plate in leucine-free media (Caisson Labs, North Logan, UT, USA) and incubated overnight at 37°C with 5% CO_2_ to allow cells to adhere. Leucine-free solutions of cytotoxins in varying concentrations were added to wells in triplicate. Incubation continued for 72 h with ^3^H-leucine (GE Healthcare, Chalfont St Giles, UK) added (1 *μ*Ci per well) for the final 24 h of incubation. Plates were frozen to detach cells and cells were then harvested onto a glass fibre filter, washed, dried, and counted using standard scintillation methods. Data from protein synthesis assays are reported as percentage of control (media alone) c.p.m./20 000 cells. IC_50_ values indicate the concentration of cytotoxin required to inhibit protein synthesis by 50% compared with media alone.

Effect of EGF4KDEL on HPB-MLT cells was determined using a proliferation assay measuring ^3^H-thymidine incorporation. For this assay, cells (2 × 10^4^ per well) were mixed with cytotoxins and incubation continued for 72 h. Methyl-[^3^H]-thymidine (GE Healthcare) at a concentration of1 *μ*Ci per well was added for the final 8 h of incubation. Data collection and analysis proceeded as described above.

Blocking studies were conducted to test the functionality of both the EGF and IL-4 ligands on the EGF4KDEL molecule. Briefly, 10 nM of concentrations of either recombinant EGF and IL-4 (manufactured as described above) or a combination of both was added to leucine-free media containing 0.01 nM EGF4KDEL. Resulting mixtures were added to wells containing H513 cells and protein synthesis was measured as described above. Here, 2219, a recombinant bispecific scFv targeting the CD22 and CD19 B-cell surface molecules was included as a negative control (Vallera *et al*, 2005b).

### Flow cytometry analysis of EGF4KDEL binding

To measure binding to MM and normal cell lines, EGF4KDEL and AHN-12 (negative control) were labelled with fluorescein isothiocyanate (FITC) as described previously (Stish *et al*, 2007a). FITC-labeled proteins at a final concentration 1000 nM were incubated with 1 × 10^6^ cells in 100 *μ*l of PBS+2% FBS on ice for 30 min to allow binding. Following incubation cells were washed three times and binding was measured using FACSCalibur and CellQuest software (BD Biosciences, San Jose, CA, USA). Mean fluorescence index (MFI) of EGF4KDEL-specific binding was determined by subtracting the mean fluorescence of cells incubated with AHN-12 from that of cells incubated with EGF4KDEL.

### Determining immunogenicity of EGF4KDEL and EGF4KDEL 7Mut in Balb/c mice

Mouse immunisation studies were utilised to determine whether EGF4KDEL 7Mut elicited less of an immune response than the original EGF4KDEL molecule. Female Balb/c mice (*n*=6/group) were injected once weekly with 1 *μ*g of either EGF4KDEL or EGF4KDEL 7Mut for 4 weeks. Five days after the final injection mice were euthanized and blood was collected via cardiac puncture. Serum from each mouse was isolated using centrifugation and frozen. The amount of anti-PE38KDEL IgG in each serum sample was measured using indirect ELISA. Briefly, 5 *μ*g of purified recombinant PE38KDEL was added to each well of a 96-well microtiter plate and adhered overnight at 4°C. Unbound protein was washed away with PBS-T and blocking was performed for 1 h with 5% milk/PBS-T. Serum samples were diluted in 1 : 10 000 and 100 *μ*l of each was added to appropriate wells in triplicate. Following 3 h incubation, each well was washed 3 × with PBS-T. Peroxidase-conjugated rabbit anti-mouse IgG (Sigma) was added to each well for a 2 h room temperature incubation. Following washing *o*-Phenylenediamine dihydrochloride substrate was added to each well. After 30 min, the absorbance at 490 nm was measured for each well using a microplate reader. Quantitation of actual anti-PE38KDEL IgG present in each sample was determined by comparing the absorbance values in each well to a standard curve prepared using Protein-A-purified mouse anti-PE38KDEL.

### *In vivo* efficacy of EGF4KDEL and EGF4KDEL 7Mut against xenograft models of peritoneal malignant mesothelioma

Male *nu/nu* mice were purchased from the National Cancer Institute, Frederick Cancer Research and Development Center, Animal Production Area and housed in an Association for Assessment and Accreditation of Laboratory Animal Care-accredited specific pathogen-free facility under the care of the Department of Research Animal Resources, University of Minnesota. Animal research protocols were approved by the University of Minnesota Institutional Animal Care and Use Committee. All animals were housed in microisolator cages to minimise the potential of horizontal contamination.

Mice were imaged in real time and images were captured using Xenogen Ivis imaging system (Xenogen Corporation, Hopkington, MA, USA) and analysed IGOR Pro 4.09a software (WaveMetrics Inc., Portaland, OR, USA). Before imaging, mice were anaesthetised using isofluorane gas. All mice received 100 *μ*l of a 30 mg ml^−1^ D-luciferin aqueous solution (Gold Biotechnology, St Louis, MO, USA) as a substrate for luciferase 10 min before imaging. All images represent a 5 min exposure time and all regions of interest are expressed in units of photons/sec/cm^2^/sr.

For this study, we modified two previously established xenograft models of MM (Smythe *et al*, 1994; Frizelle *et al*, 2000). In experiment one, intraperitoneal Mesothelioma tumours were established by injecting mice with 2 × 10^6^ H2373/Luc/GFP cells suspended in 0.5 ml PBS on day 0. On day 14, luciferase activity was measured in each mouse to confirm tumour development and tumour-bearing mice were randomized into either no treatment or EGF4KDEL treatment groups (*n*=6/group). Mice in the treatment group were given daily 4 *μ*g injections of EGF4KDEL in 0.1 ml PBS on days 14,15,16 and treatment was continued QOD from day 20 through 33 (a total of 10 injections given). All treatments were administered intraperitoneally. Mice were imaged weekly to monitor tumour growth. On day 43, mice in both no treatment and EGF4KDEL groups were euthanized and GFP-expressing tumour colonies in the peritoneum were photographed as an additional measure of tumour burden. Fluorescent images were recorded using a Maestro Imaging Station (CRI, Woburn, MA, USA). Two blinded parties independently determined the number of GFP-positive tumour colonies on two separate viewings of the peritoneal cavity and the average of these independent counts were reported.

A second murine xenograft model of intraperitoneal Mesothelioma was established using the H513/Luc/GFP cell line. For experiment two, tumours were initiated by injecting a 1.0 ml solution of PBS containing 2 × 10^6^ H513/Luc/GFP cells into the peritoneum on day 0. Luciferase imaging was conducted as described above. On day 16 tumour-bearing mice were divided into either a no treatment or EGF4KDEL 7Mut treatment group (*n*=9/group). Mice in the treatment group were given 4 *μ*g injections of EGF4KDEL 7Mut. The first course of treatment with EGF4KDEL 7mut commenced on day 16 and consisted of an IP injection of 4 *μ*g each day for 4 days MTWTh. Additional courses commenced on days 22, 28, 38, 45, 59, 65, 71, 78, 86, 92, 99, 107, 134, and 142. Luciferase imaging was conducted weekly to monitor treatment efficacy.

### Statistical analyses

All statistical analysis was performed using Prism 4 (Graphpad Software, San Diego, CA, USA). Groupwise comparisons of single data points were made by Student's *t*-test. *P*-values <0.05 were considered significant.

## Results

### *In vitro* efficacy of EGF4KDEL against human MM cells

The ability of EGF4KDEL to kill cultured human MM cells was determined using the EGFR^+^ and IL-4R^+^ H2373 cells in a protein synthesis inhibition assay. Figure 1A shows that EGF4KDEL had impressive activity against H2373 as indicated by IC_50_ value of 1.3 × 10^−5^ nM. This assay also shows that the bispecific EGF4KDEL was more potent when compared with the monospecific cytotoxins EGFKDEL (IC_50_=4.3 × 10^−5^) and IL4KDEL (IC_50_=4.0 × 10^−3^ nM) targeting EGFR or IL-4R, respectively. BIC3KDEL, a PE38(KDEL)-containing agent targeting the T-cell-specific CD3 molecule, had no effect on H2373 cells revealing the specificity of toxin-mediated cell killing. Figure 1B shows that similar cytotoxicity was measured against five different MM cell lines in separate assays. Interestingly, EGF4KDEL displayed significant anti-cancer activity towards cell lines classified in both the epithelioid and sarcomatoid morphological subtypes of MM. These data show the *in vitro* cytotoxicity of EGF4KDEL towards a range of human MM cell lines.

### Specificity of EGF4KDEL

To show EGF4KDEL specificity, we incubated EGFR^−^ and IL-4R^−^ HBP-MLT leukaemic T cells with varying concentrations of both EGF4KDEL and BIC3KDEL. Figure 1C shows that while BIC3KDEL possesses excellent cytotoxicity towards these CD3^+^ cells, EGF4KDEL had no effect on cell proliferation. *In vitro* blocking assays were also conducted to show that both the EGF and IL-4 molecules contribute to EGF4KDEL activity. Figure 1D reveals that the activity of 0.01 nM EGF4KDEL towards H513 cells is only minimally decreased in the presence of either 10 nM recombinant EGF or IL-4 alone. Probably, the blocking of only one of the two ligand leaves the other available to bind and kill. However, when both EGF and IL-4 are added in combination, H513 cell killing was completely abolished. Conversely, adding a similar concentration of the non-binding 2219 recombinant molecule had no effect on the ability of EGF4KDEL to inhibit H513 cell protein synthesis. Additional studies were performed and anti-EGFR or IL-4R antibodies were used to block EGFRKDEL activity. Each partially blocked (Oh *et al*, 2009). These results show that both the EGF and IL-4 ligands specifically target their respective receptors and each ligand contributes individually to the binding and subsequent cytotoxicity of EGF4KDEL.

### Binding of EGF4KDEL to MM cells compared with normal mesothelium

**Figure 2:**
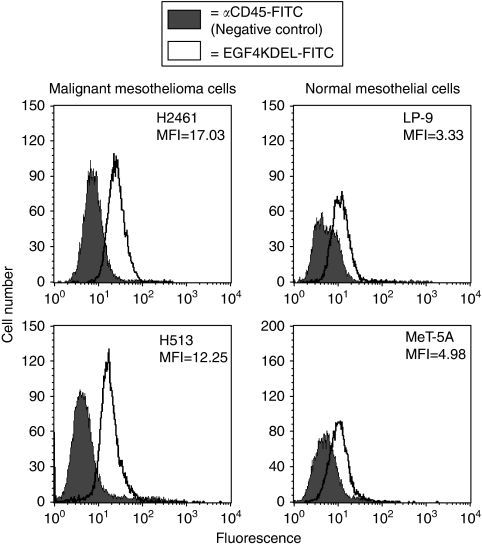
Binding of EGF4KDEL to malignant and normal Mesothelial cells. The binding of EGF4KDEL to malignant mesothelioma (H2461 and H513) and normal noncancerous mesothelial (LP-9 and MeT-5A) cell lines was measured using flow cytometry. Cells were incubated with FITC-labeled EGF4KDEL and analysed using a FACSCalibur. Histograms show the measured fluorescence of cells incubated with EGF4KDEL (no fill) compared with those exposed to a control anti-CD45-FITC antibody (grey fill). Mean fluorescence index (MFI) values represent the median fluorescence of EGF4KDEL binding minus background fluorescence measured with the negative control.

One advantage ascribed to BLT over traditional chemotherapeutics is their ability to target and kill malignant cells while sparing normal tissue. As all BLT cytotoxicity is dependent on cell surface binding and internalisation, we measured the ligand-mediated binding to both malignant mesothelioma and normal mesothelial cells. Cell lines were incubated with a 1000 nM solution of FITC-labeled EGF4KDEL and subsequently analysed using flow cytometry. Figure 2 shows a lower level of binding to LP-9 and MeT-5A normal mesothelial cells with an MFI of 3.33 and 4.98, respectively. On the other hand, stronger EGF4KDEL binding to malignant H2461 cells (MFI=17.03) was observed, representing a greater than fivefold increase over LP-9 binding. Similarly, H513 cells show a 3.7 times greater level of EGF4KDEL binding (MFI of 12.25) over non-cancerous LP-9 cells. These observations reveal greater levels of EGF4KDEL binding to MM cells with overexpressed EGFR and IL-4R compared with normal mesothelial cells.

### *In vivo* toxicity of EGF4KDEL

To determine whether a BLT linking both targeting ligands on the same single-chain molecule had any toxicity advantages over treating mice with a mixture of the two monospecific agents, survival of normal mice given varying amounts of EGF4KDEL was compared with survival of mice given similar doses of an equimolar mixture of monomeric EGFKDEL and IL4KDEL. Table 1 shows the advantages of having both ligands on the same single-chain molecule evidenced by the fact that we were able to give significantly higher doses of the BLT than a mixture of the monomeric IL4KDEL and EGFKDEL. Although half of the mice treated with two 0.5 *μ*g injections of the EGFKDEL/IL4KDEL combination did not survive, all mice receiving 2 *μ*g of EGF4KDEL survived without signs of toxicity. In fact, since further studies revealed that 4 *μ*g injections were also tolerated without weight loss, subsequent efficacy experiments were performed using a 4 *μ*g multiple-dose regimen representing a nearly 10-fold decrease toxicity compared with the mixture of monospecific agents. The lack of toxicity from EGF4KDEL treatment is especially important in a partial on-target model where the human EGF ligand cross-reacts with native murine EGFR (Nakagawa *et al*, 1985).

### Efficacy of EGF4KDEL against intraperitoneal H2373 xenografts

**Figure 3:**
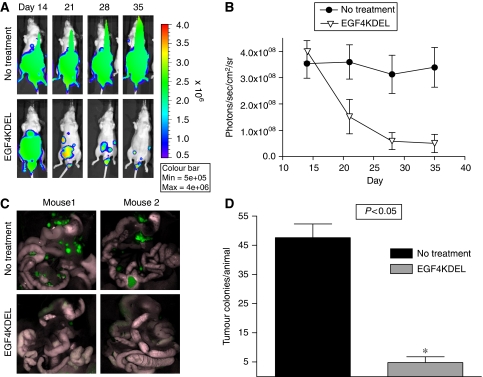
Efficacy of EGF4KDEL against intraperitoneal H2373 tumours. (**A**) Images showing the luciferase activity of H2373/Luc/GFP tumours in individual mice throughout the course of the experiment. Pictures in each row show the weekly luciferase activity observed in a representative untreated mouse (top) and a representative mouse treated with EGF4KDEL (bottom), (**B**) Graph depicting the mean measured luciferase activity±s.d. from mice bearing H2373/Luc/GFP tumours treated with EGF4KDEL compared with untreated controls (*n*=6/group). Luciferase activity was measured for a set region of interest (ROI) and is reported in units of photons/sec/cm^2^/sr. Mice in the treatment group were treated with a total of 10 intraperitoneal injections of 4 *μ*g EGF4KDEL given between days 14 and 34. (**C**) Pictures showing intraperitoneal detection of GFP-positive H2373 tumours following treatment with EGF4KDEL. Each row of pictures shows representative images of peritoneal contents from two mice in either EGF4KDEL or no treatment control group. Mice in each group were euthanized following treatment to count the number of tumour colonies per animal. (**D**) Graph showing average number of tumour colonies per animal in both the treatment and control group (*n*=6/group). Tumour colonies were counted by blinded observers. Bars represent average number of colonies per animal±s.d. ^*^Comparison of averages by Student's *t*-test resulted in *P*-value <0.05.

To determine the *in vivo* activity of EGF4KDEL a bioluminescent intraperitoneal tumour model was developed. Nude mice bearing H2373 tumours stably expressing firefly luciferase and green fluorescent protein genes were treated with 10 injections of EGF4KDEL from day 14 through day 34. Figure 3A shows representative images comparing luciferase activity in one mouse treated with EGF4KDEL to an untreated control animal. These pictures reveal that EGF4KDEL decreased tumour-associated bioluminescence compared with a mouse receiving no treatment. Average luciferase activity of all mice in this experiment is shown in Figure 3B, demonstrating a significant difference (*P*<0.02) between mice receiving EGF4KDEL and untreated controls (*n*=6/group). To prove the necessity of toxin-mediated killing, additional mice were treated with EGF4, a bispecific ligand devoid of a PE38(KDEL) and the B-cell-targeting agent 2219KDEL made with the same PE38(KDEL) construct as EGF4KDEL. These animals showed no decrease in luciferase activity relative to untreated controls (data not shown) demonstrating the targeted specificity of BLT activity. One week following the completion of treatment, tumour burden was analysed in all animals by quantifying fluorescent tumour colonies as described in the Materials and Methods. Figure 3C shows photographs of the peritoneal cavities from two mice in both the EGF4KDEL and no treatment groups. Whereas the untreated animals have diffuse GFP-positive H2373 tumour colonies, minimal tumours are seen in treated mice. Two blinded independent observers counted visible tumour colonies and the results are shown in Figure 3D. EGF4KDEL mice had 90% fewer colonies with an average of 4.8 tumours compared with 47.6 tumours per animal in the untreated control group (*P*<0.05). Together, the use of two separate imaging platforms in the H2373 model revealed that EGF4KDEL possesses significant *in vivo* activity against human MM.

### Mutation of EGF4KDEL to decrease immunogenicity

**Figure 4:**
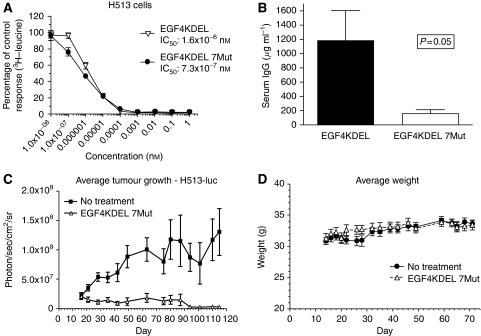
EGF4KDEL 7Mut. (**A**) H513 cells were incubated with either EGF4KDEL or EGF4KDEL 7Mut for 72 h and protein synthesis was measured. Data is reported as percent control response. Points indicate mean of triplicate measures±s.d. (**B**) Bar graph showing anti-PE38(KDEL) IgG from the serum of Balb/c mice (*n*=6/group) immunized with either EGF4KDEL or EGF4KDEL 7Mut. Measurements were made using an indirect ELISA and quantification of antibody levels was determined using a standard curve (not shown, *r*^2^>0.97) generated with a protein-purified antibody. (**C**) Graph depicting the average photons/sec/cm^2^/sr of an animal given EGF4KDEL 7Mut or untreated controls (*n*=9/group) (*P*<0.001). Data points indicate mean total bioluminescence of all mice±s.d., as determined by luciferase imaging. (**D**) Average weight of mice in EGF4KDEL 7Mut-treated and untreated control group (*n*=9/group) throughout the course of the study. Animal weights were recorded approximately three times per week to monitor any treatment-related toxicity.

To decrease the immunogenicity of EGF4KDEL, the seven major B-cell epitopes on the PE38(KDEL) portion were mutated. We tested whether these mutations affected the anti-MM activity of the molecule. Figure 4A shows that EGF4KDEL 7Mut showed impressive ability to kill H513 cells with an IC_50_ of 7.3 × 10^−7^ nM. More importantly, the activity of EGF4KDEL 7Mut was nearly identical to that of the non-mutated EGF4KDEL molecule.

Immunisation studies with immunocompetent BALB/c mice were conducted to compare the immunogenicity of EGF4KDEL 7Mut to the parental EGF4KDEL molecule. Mice (*n*=6/group) were immunized weekly for a total of 4 weeks. Serum samples from each animal were analysed using ELISA to detect anti-PE38(KDEL) IgG. Figure 4B shows that mice injected with the original EGF4KDEL agent had an average antibody concentration of 1191 *μ*g ml^−1^ compared with just 155 *μ*g ml^−1^ measured in those receiving EGF4KDEL 7Mut. Thus, mice given multiple injections of EGF4KDEL 7Mut generated 87% fewer neutralising antibodies than those injected with an equal amount of parental EGF4KDEL (*P*=0.05).

### Treatment of intraperitoneal H513 tumours with EGF4KDEL 7Mut

**Figure 5:**
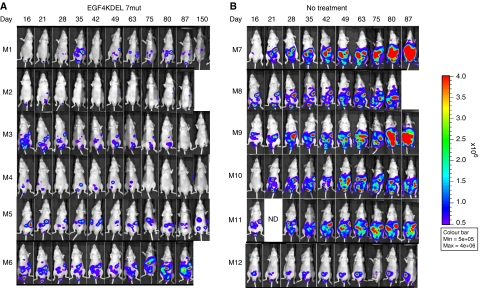
*In vivo* activity of EGF4KDEL 7Mut against H513 tumours. Photographs of representative mice showing measured luciferase activity of H513/Luc/GFP tumours in both (**A**) EGF4KDEL 7mut treatment group and (**B**) no treatment groups.

EGF4KDEL 7Mut was tested in a second xenograft MM model. Intraperitoneal tumours were induced by injecting nude mice with a 1.0 ml suspension of 2 × 10^6^ H513/Luc/GFP cells. Mice bearing established tumours were either injected with EGF4KDEL 7Mut or left untreated (*n*=9/group). The average tumour-associated luciferase activity from animals in each group is shown in Figure 4C. This graph shows that treatment of mice with EGF4KDEL 7Mut significantly reduced the tumour burden compared with untreated animals (*P*<0.001). Furthermore, despite an aggressive treatment regimen in which 15 courses were administered, no treatment-related toxicity was observed, indicated by unchanged average animal weights in Figure 4D. Figure 5 shows individual tumour-associated luciferase activity from representative mice in Figure 4C throughout the course of the experiment. Although the animals in the untreated control group display clear tumour growth and progression through day 87, mice receiving EGF4KDEL 7Mut show an excellent response to therapy indicated by markedly reduced bioluminescence. The bioluminescent imaging findings were correlated with histology studies (H&E-stained tissue samples) taken from all long-term survivors (day 150). Examination of liver, kidney, gut, spleen, and lung samples revealed excellent correlation between histological tumour reduction and levels of bioluminescence supporting the validity of this imaging model.

## Discussion

The original contribution of this research is the development of EGF4KDEL 7Mut, a promising new anti-mesothelioma agent with potential for clinical development. By linking EGF and IL-4, two well-known tumour-targeting ligands, to a truncated pseudomonas exotoxin A molecule, we created a novel recombinant agent that showed potent *in vitro* and *in vivo* activity. EGF4KDEL showed receptor-specific picomolar activity against a panel of human MM cell lines and also induced an impressive decrease in tumour burden in an aggressive H2373 intraperitoneal xenograft model. Importantly, through genetic modification we were able to create EGF4KDEL 7Mut, a second generation BLT with nearly 90% less immunogenicity that showed impressive *in vivo* potency against a highly aggressive and difficult to treat H513 xenograft model (Frizelle *et al*, 2000). In addition, toxicity was diminished by having both ligands on the same single-chain molecule allowing administration of significantly greater doses of the BLT than a mixture of the monomeric IL4KDEL and EGFKDEL. These improvements address major obstacles in the use of biological drugs and will permit multiple dosings in future clinical studies increasing the odds of anti-tumour efficacy.

In order for targeted toxins to become viable treatment options for solid tumour, steps must be taken to decrease their immunogenicity so that multiple treatments can be given to sustain high enough serum levels of drug to penetrate the tumour (Pirker, 1988). Recent clinical trials testing PE38-based anti-cancer agents for the treatment of mesothelioma and glioma revealed that 73 and 88% of the patients respectively developed neutralising IgG antibodies, with one study showing the majority of the antibodies were directed to the toxin fragment of the molecule (Hassan *et al*, 2007; Vogelbaum *et al*, 2007). To address this issue, Onda and Pastan used serum samples from patients receiving a BL22, a PE38-containing anti-leukemia agent, to map out regions of the molecule that elicited the strongest antibody response (Onda *et al*, 2006). Upon identifying seven major reactive epitopes, they proved that mutations could be made to these regions without compromising tumour cell killing (Onda *et al*, 2008). Thus, we eliminated the same seven epitopes on our EGF4KDEL molecule using site-directed mutagenesis. Studies revealed that this new agent, EGF4KDEL 7Mut, retained the same potent anti-MM activity of the original molecule. Even more impressively, mice immunized with EGF4KDEL 7Mut showed a greater than 80% reduction in anti-PE38 neutralising antibodies. Additional studies suggest that mice receiving EGF4KDEL 7Mut generate very little immune response to the binding portion of the molecule. When the ELISA was run against the entire EGF4KDEL molecule rather than against only the toxin, IgG levels were similar (data not shown). The same would be expected clinically as both EGF and IL-4 are native human cytokines.

One important issue is whether the mouse is a valid model for patient anti-toxin responses. Onda and Pastan have tested sera from patients receiving non-mutated PE38-targeted toxins and found that their anti-PE38 antibodies bind to the same seven B-cell epitopes defined by the mouse studies and eliminated in EGF4KDEL 7mut (Onda *et al*, 2008). This implies that the mouse model correlates directly to the human antibody response and is highly useful for immunogenicity studies.

One major advantage offered by recombinant anti-cancer agents is that their structure and biological activity can be altered. The ultimate goal of decreasing the immunogenicity of EGF4KDEL was to improve its pharmacokinetic profile as its efficacy is enhanced with prolonged exposure to tumour cells, as evidenced by the advantage of multiple dosings. Formation of neutralising antibodies leads to rapid serum clearance of the drug and precludes the opportunity to administer additional doses. Conjugation of polyethylene glycol to targeted toxins has been used as a strategy to reduce immunogenicity; however, this strategy often leads to a loss of catalytic activity (Filpula *et al*, 2007). The direct genetic modification used in this study eliminates the additional step of PEGylation and retains the full cytotoxic capability of parental molecule. Other studies have shown that using genetic engineering to mutate T-cell-specific epitopes and the proteolytic cleavage site can improve the pharmacokinetic profile and *in vivo* efficacy of the biological drug (Tangri *et al*, 2005; Weldon *et al*, 2008). Additional studies may show that incorporation of similar mutations into EGF4KDEL 7Mut can further enhance its already potent anti-tumour activity.

Another issue facing nearly every anti-cancer chemotherapeutic agent is treatment-related toxicity. BLT is designed to specifically target surface receptors that are overexpressed on cancer cells, thereby minimising toxic effects to normal tissue. We have shown that EGF4KDEL's cytotoxicity is receptor specific as EGFR-/IL-4R- HPB-MLT leukaemia cells are unaffected by concentrations as high as 10 nM. The binding studies reveal that MM cell lines have 3- to 5-fold higher levels of EGF4KDEL bound to their surface than normal human mesothelial cells. This binding preference of EGF4KDEL for malignant cells was also observed in protein synthesis inhibition assays. Whereas exposure to 100 fM concentrations of EGF4KDEL inhibited more than 90% of H513 cell protein synthesis, the anabolic activity of normal Met-5A cells incubated with the same amount of BLT was decreased by less than 20% (data not shown). Specific targeting is further evidenced in *in vivo* studies by the fact that 18 intraperitoneal injections of EGF4KDEL 7Mut yielded no toxicity (Figure 5C) in a mouse model where the human EGF molecule cross-reacts with the murine EGFR (Nakagawa *et al*, 1985). Although human IL-4 does not cross-react with murine IL-4R (Mosmann *et al*, 1987), Table 1 shows that its presence on EGF4KDEL results in a significantly improved toxicity profile compared with monospecific EGFKDEL. This decrease in toxicity may be due to the fact that the larger BLT molecule exceeds the glomerular basement membrane filtration capacity, resulting in decreased nephrotoxicity. Further pharmacokinetic studies with a full on-target model, such as non-human primates, would be necessary to fully elucidate the toxicity benefits of EGF4KDEL 7Mut compared with the monospecific EGFKDEL and IL4KDEL. Taken together, these studies reveal that the ligand-directed targeting of EGF4KDEL 7Mut allows maximal MM cell killing and may minimise collateral toxicity to healthy tissues.

This research shows for the first time that targeting of EGFR and IL-4R simultaneously with a BLT is a promising therapeutic option in the treatment of MM. Figure 1A reveals that targeting both receptors simultaneously with EGF4KDEL leads to more potent cell killing than targeting either receptor alone. However, the activity of this agent does not require concomitant overexpression of both EGFR and IL-4R. As shown by Figure 1D, both the EGF and IL-4 ligands can contribute individually to the activity of EGF4KDEL, thus broadening the utility of this agent, allowing it to target tumours expressing varying amounts of EGFR or IL-4R in combination or individually.

Despite the fact that greater than 75% of MM tumour samples overexpressed EGFR, clinical trials targeting EGFR in MM with tyrosine kinase inhibitors have yielded some objective responses, but not with the desired frequency (Govindan *et al*, 2005; Garland *et al*, 2007). This inconsistency of response is likely because of the fact that few MM tumours possess the activating EGFR mutations that correlate with tyrosine kinase inhibitor efficacy (Cortese *et al*, 2006). With the mechanism EGF4KDEL cell killing depending on ligand-mediated binding and internalisation, EGFR mutation status should not affect BLT-mediated anti-MM activity. Although targeting of IL-4R in MM has not yet been pursued clinically, expression studies showed moderate to strong staining in 13 of 13 patient-tumour samples, proving it to be a logical therapeutic target (Beseth *et al*, 2004). IL-4R is an ideal candidate for targeted toxin therapy because of its rapid internalisation upon ligand binding and limited expression profile in normal tissues (Kawakami *et al*, 2002a, 2002b). Numerous preclinical studies have shown the potent anti-tumour activity of a monospecific cytotoxin composed of IL-4 linked to PE38 (Kreitman *et al*, 1994; Shimamura *et al*, 2007). However, efficacy of this agent in a Phase I clinical trial of patients with renal cell carcinoma and NSCLC yielded no objective tumour responses, likely because of the rapid development of neutralising antibodies (Garland *et al*, 2005).

Another important aspect of these studies is the use of *in vivo* tumour imaging to assess the therapeutic efficacy of novel BLT in real time against intraperitoneal models of MM and the observation of long-term anti-MM affects. Traditionally, treatment outcomes in intraperitoneal xenograft models of MM were only able to be determined using expensive long-term survival studies or by euthanizing animals to count tumour colonies (Frizelle *et al*, 2000; Stewart *et al*, 2002). By introducing the firefly luciferase gene into H2373 and H513 we were able to monitor the effect of treatment on tumour burden on a weekly basis using a highly sensitive and well-validated bioluminescent imaging technology that directly correlates with actual tumour volume (Jenkins *et al*, 2003). Furthermore, using H2373 cells stably expressing green fluorescent protein for our *in vivo* study, we were able to accurately assess and quantitate peritoneal tumour colonies at the completion of treatment. This strategy has been shown to be much more sensitive for detecting and assessing tumour nodules (Udagawa *et al*, 2002; Oh *et al*, 2009).

The murine model of intraperitoneal human MM is currently considered the appropriate *in vivo* model for studying localised therapy. Despite the fact that only about 11% of clinical mesothelioma cases are confined to the peritoneum (Hassan *et al*, 2006), the model is also considered a well-accepted surrogate for the more common pleural mesothelioma. Although some MM studies have shown promise for localised intraperitoneal and intrapleural administration of chemotherapeutics (Hotta *et al*, 2004; Sterman *et al*, 2007), most trials have been inconclusive. Localised administration of EGF4KDEL may offer advantages in lower systemic toxicity, eased immune response, and greater tumour targeting. Systemic therapy is currently the standard of care for treatment of MM. A recent Phase I trial showed that systemic administration of SS1P, a recombinant-targeted toxin similar to EGF4KDEL 7Mut, resulted in stable disease or minor responses in 14 of 19 patients with MM (Hassan *et al*, 2007). Our *in vivo* experiments were conducted with intraperitoneal injection of EGF4KDEL 7Mut because of the technical difficulties associated with frequent multiple i.v. injections in mice. These data reveal that intraperitoneal injection of BLT was highly effective at decreasing tumour burden in two separate aggressive models of MM where treatment started 14–16 days post tumour inoculation; however, further experiments will be needed to compare local infusion of EGF4KDEL 7Mut to systemic therapy.

In conclusion, EGF4KDEL 7Mut is a powerful new alternative therapy for drug refractory MM. Our *in vitro* studies show this drug exhibits potent and selective activity. More importantly, we reduced *in vivo* drug toxicity and by introducing a series of amino-acid substitutions, we were able to decrease the immunogenicity of EGF4KDEL 7Mut and ultimately improve its potential for use in a clinical setting for MM therapy.

## Figures and Tables

**Table 1:** The effect of EGF4KDEL7 on animal mortality

	**Deaths/total no. of mice**			
**Drug (*μ*g)**	**2**	**1**	**0.5**	**0.25**
EGFKDEL	4/4	3/4	1/4	0/4
IL4KDEL	0/4	0/4	—	—
EGFKDEL+IL4KDEL	6/6	4/4	2/4	0/4
EGF4KDEL	0/4	0/4	—	—

Normal C57BL/6 mice received an i.p. injection of drug and then 2 days later, a second injection was given. Toxic deaths occurred within 7 days. Animals were observed for 3 weeks.

## References

[bib1] Beseth BD, Cameron RB, Leland P, You L, Varricchio F, Kreitman RJ, Maki RA, Jablons DM, Husain SR, Puri RK (2004) Interleukin-4 receptor cytotoxin as therapy for human malignant pleural mesothelioma xenografts. Ann Thorac Surg 78: 436–443 15276492 10.1016/j.athoracsur.2004.03.010

[bib2] Cole Jr GW, Alleva AM, Reddy RM, Maxhimer JB, Zuo J, Schrump DS, Nguyen DM (2005) The selective epidermal growth factor receptor tyrosine kinase inhibitor PD153035 suppresses expression of prometastasis phenotypes in malignant pleural mesothelioma cells *in vitro*. J Thorac Cardiovasc Surg 129: 1010–1017 15867774 10.1016/j.jtcvs.2004.10.040

[bib3] Cortese JF, Gowda AL, Wali A, Eliason JF, Pass HI, Everson RB (2006) Common EGFR mutations conferring sensitivity to gefitinib in lung adenocarcinoma are not prevalent in human malignant mesothelioma. Int J Cancer 118: 521–522 16052537 10.1002/ijc.21271

[bib4] Filpula D, Yang K, Basu A, Hassan R, Xiang L, Zhang Z, Wang M, Wang QC, Ho M, Beers R, Zhao H, Peng P, Zhou J, Li X, Petti G, Janjua A, Liu J, Wu D, Yu D, Zhang Z, Longley C, FitzGerald D, Kreitman RJ, Pastan I (2007) Releasable PEGylation of mesothelin targeted immunotoxin SS1P achieves single dosage complete regression of a human carcinoma in mice. Bioconjug Chem 18: 773–784 17346030 10.1021/bc060314x

[bib5] Frizelle SP, Rubins JB, Zhou JX, Curiel DT, Kratzke RA (2000) Gene therapy of established mesothelioma xenografts with recombinant p16INK4a adenovirus. Cancer Gene Ther 7: 1421–1425 11129284 10.1038/sj.cgt.7700241

[bib6] Garland L, Gitlitz B, Ebbinghaus S, Pan H, de Haan H, Puri RK, Von Hoff D, Figlin R (2005) Phase I trial of intravenous IL-4 pseudomonas exotoxin protein (NBI-3001) in patients with advanced solid tumors that express the IL-4 receptor. J Immunother 28: 376–381 16000956 10.1097/01.cji.0000162782.86008.ml

[bib7] Garland LL, Rankin C, Gandara DR (2007) Phase II study of erlotinib in patients with malignant pleural mesothelioma: a Southwest Oncology Group Study. J Clin Oncol 25: 2406–2413 17557954 10.1200/JCO.2006.09.7634

[bib8] Govindan R, Kratzke RA, Herndon II JE, Niehans GA, Vollmer R, Watson D, Green MR, Kindler HL, Cancer and Leukemia Group B (CALGB 30101) (2005) Gefitinib in patients with malignant mesothelioma: a phase II study by the Cancer and Leukemia Group B. Clin Cancer Res 11: 2300–2304 15788680 10.1158/1078-0432.CCR-04-1940

[bib9] Haigler H, Ash JF, Singer SJ, Cohen S (1978) Visualization by fluorescence of the binding and internalization of epidermal growth factor in human carcinoma cells A-431. Proc Natl Acad Sci USA 75: 3317–3321 356052 10.1073/pnas.75.7.3317 PMC392766

[bib10] Hassan R, Alexander R, Antman K, Boffetta P, Churg A, Coit D, Hausner P, Kennedy R, Kindler H, Metintas M, Mutti L, Onda M, Pass H, Premkumar A, Roggli V, Sterman D, Sugarbaker P, Taub R, Verschraegen C (2006) Current treatment options and biology of peritoneal mesothelioma: meeting summary of the first NIH peritoneal mesothelioma conference. Ann Oncol 17: 1615–1619 16600983 10.1093/annonc/mdl060

[bib11] Hassan R, Bullock S, Premkumar A, Kreitman RJ, Kindler H, Willingham MC, Pastan I (2007) Phase I study of SS1P, a recombinant anti-mesothelin immunotoxin given as a bolus I.V. infusion to patients with mesothelin-expressing mesothelioma, ovarian, and pancreatic cancers. Clin Cancer Res 13: 5144–5149 17785569 10.1158/1078-0432.CCR-07-0869

[bib12] Hassan R, Williams-Gould J, Watson T, Pai-Scherf L, Pastan I (2004) Pretreatment with rituximab does not inhibit the human immune response against the immunogenic protein LMB-1. Clin Cancer Res 10: 16–18 14734446 10.1158/1078-0432.ccr-1160-3

[bib13] Hotta T, Taniguchi K, Kobayashi Y, Johata K, Sahara M, Naka T, Tanimura H, Tsubota YT (2004) Chemotherapy and serum hyaluronic acid levels in malignant peritoneal mesothelioma. Hepatogastroenterology 51: 1073–1083 15239250

[bib14] Jenkins DE, Oei Y, Hornig YS, Yu SF, Dusich J, Purchio T, Contag PR (2003) Bioluminescent imaging (BLI) to improve and refine traditional murine models of tumor growth and metastasis. Clin Exp Metastasis 20: 733–744 14713107 10.1023/b:clin.0000006815.49932.98

[bib15] Kawakami K, Kawakami M, Leland P, Puri RK (2002a) Internalization property of interleukin-4 receptor chain increases cytotoxic effect of interleukin-4 receptor-targeted cytotoxin in cancer cells. Clin Cancer Res 8: 258–266 11801567

[bib16] Kawakami M, Kawakami K, Stepensky VA, Maki RA, Robin H, Muller W, Husain SR, Puri RK (2002b) Interleukin 4 receptor on human lung cancer: a molecular target for cytotoxin therapy. Clin Cancer Res 8: 3503–3511 12429641

[bib17] Kreitman RJ, Pastan I (1995) Importance of the glutamate residue of KDEL in increasing the cytotoxicity of Pseudomonas exotoxin derivatives and for increased binding to the KDEL receptor. Biochem J 307: 29–37 7717988 10.1042/bj3070029 PMC1136741

[bib18] Kreitman RJ, Pastan I (1998) Accumulation of a recombinant immunotoxin in a tumor *in vivo*: fewer than 1000 molecules per cell are sufficient for complete responses. Cancer Res 58: 968–975 9500458

[bib19] Kreitman RJ, Puri RK, Pastan I (1994) A circularly permuted recombinant interleukin 4 toxin with increased activity. Proc Natl Acad Sci USA 91: 6889–6893 8041715 10.1073/pnas.91.15.6889 PMC44303

[bib20] Mosmann TR, Yokota T, Kastelein R, Zurawski SM, Arai N, Takebe Y (1987) Species-specificity of T cell stimulating activities of IL 2 and BSF-1 (IL 4): comparison of normal and recombinant, mouse and human IL 2 and BSF-1 (IL 4). J Immunol 138: 1813–1816 3493289

[bib21] Nakagawa S, Yoshida S, Hirao Y, Kasuga S, Fuwa T (1985) Biological effects of biosynthetic human EGF on the growth of mammalian cells *in vitro*. Differentiation 29: 284–288 3878309 10.1111/j.1432-0436.1985.tb00328.x

[bib22] Oh S, Stish BJ, Sachdev D, Chen C, Dudek AZ, Vallera DA (2009) A novel reduced immunogenicity bispecific targeted toxin simultaneously recognizing human EGF and IL-4 receptors in a mouse model of metastatic breast carcinoma. Clin Cancer Res (e-pub ahead of print 1 October 2009) 10.1158/1078-0432.CCR-09-0696 PMC2756320 19789305

[bib23] Onda M, Beers R, Xiang L, Nagata S, Wang QC, Pastan I (2008) An immunotoxin with greatly reduced immunogenicity by identification and removal of B cell epitopes. Proc Natl Acad Sci USA 105: 11311–11316 18678888 10.1073/pnas.0804851105 PMC2516223

[bib24] Onda M, Nagata S, FitzGerald DJ, Beers R, Fisher RJ, Vincent JJ, Lee B, Nakamura M, Hwang J, Kreitman RJ, Hassan R, Pastan I (2006) Characterization of the B cell epitopes associated with a truncated form of Pseudomonas exotoxin (PE38) used to make immunotoxins for the treatment of cancer patients. J Immunol 177: 8822–8834 17142785 10.4049/jimmunol.177.12.8822

[bib25] Palumbo C, Bei R, Procopio A, Modesti A (2008) Molecular targets and targeted therapies for malignant mesothelioma. Curr Med Chem 15: 855–867 18473795 10.2174/092986708783955446

[bib26] Pass HI, Stevens EJ, Oie H, Tsokos MG, Abati AD, Fetsch PA, Mew DJ, Pogrebniak HW, Matthews WJ (1995) Characteristics of nine newly derived mesothelioma cell lines. Ann Thorac Surg 59: 835–844 7695406 10.1016/0003-4975(95)00045-m

[bib27] Pastan I, Chaudhary V, FitzGerald DJ (1992) Recombinant toxins as novel therapeutic agents. Annu Rev Biochem 61: 331–354 1497314 10.1146/annurev.bi.61.070192.001555

[bib28] Pirker R (1988) Immunotoxins against solid tumors. J Cancer Res Clin Oncol 114: 385–393 3045130 10.1007/BF02128183 PMC12243822

[bib29] Rand RW, Kreitman RJ, Patronas N, Varricchio F, Pastan I, Puri RK (2000) Intratumoral administration of recombinant circularly permuted interleukin-4-*Pseudomonas* exotoxin in patients with high-grade glioma. Clin Cancer Res 6: 2157–2165 10873064

[bib30] Shimamura T, Royal RE, Kioi M, Nakajima A, Husain SR, Puri RK (2007) Interleukin-4 cytotoxin therapy synergizes with gemcitabine in a mouse model of pancreatic ductal adenocarcinoma. Cancer Res 67: 9903–9912 17942922 10.1158/0008-5472.CAN-06-4558

[bib31] Simon GR, hraegen CF, Jänne PA, Langer CJ, Dowlati A, Gadgeel SM, Kelly K, Kalemkerian GP, Traynor AM, Peng G, Gill J, Obasaju CK, Kindler HL (2008) Pemetrexed plus gemcitabine as first-line chemotherapy for patients with peritoneal mesothelioma: final report of a phase II trial. J Clin Oncol 26: 3567–3572 18640937 10.1200/JCO.2007.15.2868

[bib32] Smythe WR, Kaiser LR, Hwang HC, Amin KM, Pilewski JM, Eck SJ, Wilson JM, Albelda SM (1994) Successful adenovirus-mediated gene transfer in an *in vivo* model of human malignant mesothelioma. Ann Thorac Surg 57: 1395–1401 8010779 10.1016/0003-4975(94)90090-6

[bib33] Sterman DH, Recio A, Carroll RG, Gillespie CT, Haas A, Vachani A, Kapoor V, Sun J, Hodinka R, Brown JL, Corbley MJ, Parr M, Ho M, Pastan I, Machuzak M, Benedict W, Zhang XQ, Lord EM, Litzky LA, Heitjan DF, June CH, Kaiser LR, Vonderheide RH, Albelda SM, Kanther M (2007) A phase I clinical trial of single-dose intrapleural IFN-beta gene transfer for malignant pleural mesothelioma and metastatic pleural effusions: high rate of antitumor immune responses. Clin Cancer Res 13: 4456–4466 17671130 10.1158/1078-0432.CCR-07-0403

[bib34] Stewart IV JH, Nguyen DM, Chen GA, Schrump DS (2002) Induction of apoptosis in malignant pleural mesothelioma cells by activation of the Fas (Apo-1/CD95) death-signal pathway. J Thorac Cardiovasc Surg 123: 295–302 11828289 10.1067/mtc.2002.119882

[bib35] Stish BJ, Chen H, Shu Y, Panoskaltsis-Mortari A, Vallera DA (2007a) A bispecific recombinant cytotoxin (DTEGF13) targeting human IL-13 and EGF receptors in a mouse xenograft model of prostate cancer. Clin Cancer Res 13: 6486–6493 17975161 10.1158/1078-0432.CCR-07-0938

[bib36] Stish BJ, Chen H, Shu Y, Panoskaltsis-Mortari A, Vallera DA (2007b) Increasing anticarcinoma activity of an anti-erbB2 recombinant immunotoxin by the addition of an anti-EpCAM sFv. Clin Cancer Res 13: 3058–3067 17505009 10.1158/1078-0432.CCR-06-2454

[bib37] Stish BJ, Oh S, Vallera DA (2008) Anti-glioblastoma effect of a recombinant bispecific cytotoxin cotargeting human IL-13 and EGF receptors in a mouse xenograft model. J Neurooncol 87: 51–61 18084721 10.1007/s11060-007-9499-8

[bib38] Tangri S, Mothé BR, Eisenbraun J, Sidney J, Southwood S, Briggs K, Zinckgraf J, Bilsel P, Newman M, Chesnut R, Licalsi C, Sette A (2005) Rationally engineered therapeutic proteins with reduced immunogenicity. J Immunol 174: 3187–3196 15749848 10.4049/jimmunol.174.6.3187

[bib39] Udagawa T, Fernandez A, Achilles EG, Folkman J, D’Amato RJ (2002) Persistence of microscopic human cancers in mice: alterations in the angiogenic balance accompanies loss of tumor dormancy. FASEB J 16: 1361–1370 12205027 10.1096/fj.01-0813com

[bib40] Vallera DA, Chen H, Sicheneder AR, Panoskaltsis-Mortari A, Taras EP (2009) Genetic alteration of a bispecific ligand-directed toxin targeting human CD19 and CD22 receptors resulting in improved efficacy against systemic B cell malignancy. Leuk Res 33: 1233–1242 19327829 10.1016/j.leukres.2009.02.006 PMC2738628

[bib41] Vallera DA, Shu Y, Chen H, Saluja A, Vickers SM, Buchsbaum DJ, Stish BJ (2008) Genetically designing a more potent anti-pancreatic cancer agent by simultaneously cotargeting human IL-13 and EGF receptors in a mouse xenograft model. Gut 57: 634–641 18222985 10.1136/gut.2007.137802 PMC2756191

[bib42] Vallera DA, Todhunter D, Kuroki DW, Shu Y, Sicheneder A, Panoskaltsis-Mortari A, Vallera VD, Chen H (2005a) Molecular modification of a recombinant, bivalent anti-human CD3 immunotoxin (Bic3) results in reduced *in vivo* toxicity in mice. Leuk Res 29: 331–341 15661270 10.1016/j.leukres.2004.08.006

[bib43] Vallera DA, Todhunter DA, Kuroki DW, Shu Y, Sicheneder A, Chen H (2005b) A bispecific recombinant immunotoxin, DT2219, targeting human CD19 and CD22 receptors in a mouse xenograft model of B-cell leukemia/lymphoma. Clin Cancer Res 11: 3879–3888 15897589 10.1158/1078-0432.CCR-04-2290

[bib44] Vogelbaum MA, Sampson JH, Kunwar S, Chang SM, Shaffrey M, Asher AL, Lang FF, Croteau D, Parker K, Grahn AY, Sherman JW, Husain SR, Puri RK (2007) Convection-enhanced delivery of cintredekin besudotox (interleukin-13-PE38QQR) followed by radiation therapy with and without temozolomide in newly diagnosed malignant gliomas: phase 1 study of final safety results. Neurosurgery 61: 1031–1037 18091279 10.1227/01.neu.0000303199.77370.9e

[bib45] Vogelzang NJ, Rusthoven JJ, Symanowski J, Denham C, Kaukel E, Ruffie P, Gatzemeier U, Boyer M, Emri S, Manegold C, Niyikiza C, Paoletti P (2003) Phase III study of pemetrexed in combination with cisplatin versus cisplatin alone in patients with malignant pleural mesothelioma. J Clin Oncol 21: 2636–2644 12860938 10.1200/JCO.2003.11.136

[bib46] Weldon JE, Xiang L, Chertov O, Margulies I, Kreitman RJ, Fitzgerald DJ, Pastan I (2008) A protease-resistant immunotoxin against CD22 with greatly increased activity against CLL and diminished animal toxicity. Blood 113: 3792–3800 18988862 10.1182/blood-2008-08-173195 PMC2670794

[bib47] Yarden Y, Sliwkowski MX (2001) Untangling the ErbB signalling network. Nat Rev Mol Cell Biol 2: 127–137 11252954 10.1038/35052073

[bib48] Zervos MD, Bizekis C, Pass HI (2008) Malignant mesothelioma 2008. Curr Opin Pulm Med 14: 303–309 18520263 10.1097/MCP.0b013e328302851d

[bib49] Zucali PA, Giaccone G (2006) Biology and management of malignant pleural mesothelioma. Eur J Cancer 42: 2706–2714 16989994 10.1016/j.ejca.2006.07.011

